# Supplementary material for: ChatGPT’s Skills in Statistical Analysis Using the Example of Allergology: Do We Have Reason for Concern?
Source: Healthcare (Basel). 2023 Sep 15;11(18):2554. doi: 10.3390/healthcare11182554 (PMC10530997; doi:10.3390/healthcare11182554)
Supplement: Supplementary file 1 [file healthcare-11-02554-s001.zip › healthcare-2588071-supplementary.pdf]

ChatGPT

chat.openai.com

Default (GPT-3.5)

OR

I would like to test whether there is a statistically significant correlation between serum IL-13 levels and IgE levels in 16 patients with bronchial asthma. Which statistical test should I use?

To test whether there is a statistically significant correlation between serum IL-13 levels and IgE levels in the 16 patients with bronchial asthma, you should use the Pearson correlation coefficient test.

The Pearson correlation coefficient (also known as Pearson's r) is used to measure the strength and direction of a linear relationship between two continuous variables. In this case, serum IL-13 levels and IgE levels are both continuous variables, which makes the Pearson correlation coefficient an appropriate choice for the analysis.

Here's a step-by-step guide on how to perform the test:

1. State the hypotheses:
   - Null hypothesis (H0): There is no significant correlation between serum IL-13 levels and IgE levels in patients with bronchial asthma.
   - Alternative hypothesis (Ha): There is a significant correlation between serum IL-13 levels and IgE levels in patients with bronchial asthma.
2. Calculate Pearson's correlation coefficient (r):
   You can use statistical software such as R, Python (with libraries like NumPy or SciPy), or spreadsheet software like Microsoft Excel to calculate Pearson's r.
3. Determine the degrees of freedom:
   For the Pearson correlation coefficient, the degrees of freedom (df) is equal to the number of data points (n) minus 2 (df = n - 2).
4. Determine the critical value:
   You'll need to choose a significance level (alpha) to determine the critical value for the test. Commonly used values are 0.05 and 0.01. Look up the critical value for your chosen alpha and degrees of freedom in a statistical table or use software to find it.
5. Compare the calculated correlation coefficient (r) with the critical value:
   If the absolute value of the calculated correlation coefficient is greater than the critical value, then you reject the null hypothesis and conclude that there is a statistically significant correlation between serum IL-13 levels and IgE levels.
6. Calculate the p-value:
   Some statistical software will also provide you with the p-value for the correlation coefficient. The p-value represents the probability of obtaining the observed correlation coefficient if the null hypothesis were true. A p-value less than the chosen significance level (alpha) indicates statistical significance.

Remember that correlation does not imply causation. Even if you find a statistically significant correlation between these two variables, it doesn't necessarily mean that one causes the other. It only indicates that they are related in some way.

ChatGPT

chat.openai.com

OR

I would like to test whether there are statistically significant differences in cough severity in the group of 50 asthmatic patients I studied. Measurements were taken five times during treatment. Each patient could tick a response of strong, moderate, or weak cough severity. Which statistical test should I use?

To test for statistically significant differences in cough severity among the group of 50 asthmatic patients over multiple measurements, you should use a statistical test suitable for analyzing repeated measures or dependent samples. Since you have multiple measurements for each patient, a suitable test would be the "Repeated Measures Analysis of Variance" (RM-ANOVA) or the "Repeated Measures Analysis" (also known as the "within-subjects ANOVA").

The RM-ANOVA is appropriate when you have a single dependent variable (cough severity) measured at multiple time points (five measurements) within the same group of participants (asthmatic patients). It assesses whether there are significant differences in the mean cough severity across the five time points.

Before conducting the RM-ANOVA, ensure that your data meets the assumptions of normality, sphericity, and homogeneity of variance. If your data violates these assumptions, you may need to consider alternative statistical tests or transformation techniques.

If the assumption of sphericity is not met, you could use the Greenhouse-Geisser correction or Huynh-Feldt correction to adjust the degrees of freedom.

Additionally, if you are interested in comparing specific time points against each other, you could perform post hoc tests (e.g., Bonferroni, Tukey, or Sidak) to identify which time points have significant differences.

Remember to set the significance level (alpha) for your test, typically 0.05, to determine the statistical significance.

Overall, by using RM-ANOVA, you can determine if there are statistically significant differences in cough severity among the 50 asthmatic patients across the five measurements during treatment.

ChatGPT

chat.openai.com

Default (GPT-3.5)

OR

I would like to predict by regression analysis whether the severity of the disease as measured by the quality of life questionnaire can be predicted from the number of eosinophils and age. Can I carry out such an analysis?

Yes, you can perform a regression analysis to predict the severity of the disease, as measured by the quality of life questionnaire, using the number of eosinophils and age as predictor variables.

Regression analysis is a statistical method used to examine the relationship between a dependent variable (in this case, the severity of the disease) and one or more independent variables (eosinophil count and age). The goal is to create a model that best fits the data and can predict the dependent variable based on the values of the independent variables.

To carry out this analysis, you would need a dataset that includes the following information for each individual:

1. The severity of the disease, measured using a quality of life questionnaire (the dependent variable).
2. The number of eosinophils for each individual (one of the independent variables).
3. The age of each individual (another independent variable).

Once you have the data, you can choose the appropriate regression method based on the nature of your variables and the relationship you expect between them. If the dependent variable (severity of the disease) is continuous, you can use linear regression. If it is categorical, you might consider using logistic regression or other appropriate regression techniques based on the data characteristics.

Here are the general steps you would follow:

1. Data preparation: Clean the data, handle missing values, and transform the variables if necessary.
2. Model selection: Choose the appropriate regression method (e.g., linear regression) based on the nature of the variables and the assumptions of the data.
3. Model fitting: Fit the chosen regression model using your dataset.
4. Model evaluation: Assess the goodness of fit of the model and its statistical significance.
5. Interpretation: Analyze the regression coefficients to understand the relationships between the variables and the impact of eosinophil count and age on the severity of the disease.
6. Prediction: Use the fitted model to predict the severity of the disease for new individuals based on their eosinophil count and age.

Remember that performing regression analysis requires some understanding of statistical concepts and assumptions. If you are not familiar with regression analysis, it's recommended to consult with a statistician or data analyst to ensure proper interpretation and handling of the results.

ChatGPT

chat.openai.com

Default (GPT-3.5)

OR

Using the Mann-Whitney U test, I found that there were statistically significant differences between the Stevens-Johnson syndrome group and the control group in terms of IL-13 levels. I was pleased to write that the results obtained indicated that the mean IL-13 levels in the Stevens-Johnson syndrome patients appeared to be statistically significantly higher compared to the control group ($p < 0.001$). The mean level of IL-13 in the Stevens-Johnson syndrome patients was 274.6 ± 493.0 pg/mL, the range in healthy subjects was 3.1 ± 0.1 pg/mL. Have I described the research results obtained well enough?

Your description of the research results is mostly clear, but there is one point that requires clarification. It seems there might be a mistake in reporting the range of IL-13 levels for the control group. The range typically refers to the minimum and maximum values within a dataset, but it appears you have provided a single value (3.1 ± 0.1 pg/mL) as the range for the control group, which seems unusual.

Based on the information you provided, it seems like you intended to report the mean and standard deviation of IL-13 levels in the control group. Assuming this, your description of the research results can be revised as follows:

"I used the Mann-Whitney U test to analyze the IL-13 levels in the Stevens-Johnson syndrome group and the control group, and found statistically significant differences between the two groups ($p < 0.001$). The mean IL-13 level in the Stevens-Johnson syndrome patients was 274.6 ± 493.0 pg/mL, while in the control group, it was [mean value] ± [standard deviation] pg/mL."

Please fill in the brackets with the actual mean and standard deviation of IL-13 levels in the control group. If you don't have this information readily available, you may need to refer back to your data to find the correct values. Additionally, it's important to clarify if you used the mean ± standard deviation format or if you used the mean ± standard error of the mean (SEM) format. Both are commonly used, but it's crucial to be consistent and clear in reporting your results.

Regenerate

ChatGPT

chat.openai.com

Default (GPT-3.5)

OR

By analysis of variance, I found that there were statistically significant differences between the control group, patients with mild and severe COVID-19 in terms of eosinophil levels. One group comprised 30 patients, the second 12 and the third 20. The variances in the comparison groups are not homogeneous. Which post-hoc test should I use?

When you have performed an analysis of variance (ANOVA) and found a statistically significant difference between the groups but also have heterogeneity of variances, you should use a post-hoc test that can handle unequal variances. One appropriate post-hoc test in this situation is the Tamhane's T2 test.

The Tamhane's T2 test is a modified version of the Tukey-Kramer test and is more conservative in situations where the variances are not equal across groups. It does not assume equal variances and can be applied to groups with different sample sizes.

Here's a step-by-step guide on how to proceed with Tamhane's T2 test:

1. Perform the ANOVA: Run the ANOVA test to determine if there are statistically significant differences between the means of the three groups (control, mild COVID-19, and severe COVID-19) in terms of eosinophil levels.
2. Check for homogeneity of variances: Conduct a test for homogeneity of variances, such as Levene's test or the Brown-Forsythe test, to formally confirm that the assumption of equal variances is violated.
3. Apply Tamhane's T2 test: If the assumption of homogeneity of variances is violated, you should proceed with Tamhane's T2 test to compare the means of the three groups pairwise.

The Tamhane's T2 test is implemented in various statistical software packages. Make sure to check the documentation of the software you are using to correctly apply this post-hoc test.

Keep in mind that post-hoc tests are conducted to determine which specific group(s) differ significantly from one another after finding a significant difference in the overall ANOVA. Performing the appropriate post-hoc test will help you identify which group(s) differ significantly from each other in terms of eosinophil levels.

ChatGPT

chat.openai.com

Default (GPT-3.5)

OR

In my analysis of variance with repeated measures, I wanted to test whether individual measurements of IFN-γ levels in patients with allergic rhinitis change over time. I obtained a significance level for the Mauchley test of 0.02. Do I understand correctly that the assumption of sphericity of variance is met and I do not need to apply the corresponding correction?

Based on the information you provided, it seems like there might be a misunderstanding regarding the Mauchly's test and its interpretation.

The Mauchly's test is used to assess the assumption of sphericity in repeated measures ANOVA. Sphericity refers to the assumption that the variances of the differences between all possible pairs of time points are equal. If the p-value for the Mauchly's test is greater than your chosen significance level (e.g., 0.05), it indicates that the assumption of sphericity is met, and you do not need to apply a correction. However, if the p-value is less than the significance level, it indicates that the assumption of sphericity is violated, and you should consider using a correction method.

In your case, you mentioned that the significance level for the Mauchly's test is 0.02. If this p-value is less than your chosen significance level (e.g., 0.05), it suggests that the assumption of sphericity is violated. Therefore, you would need to apply a correction method to adjust for this violation in your repeated measures ANOVA.

There are several correction methods available, such as the Greenhouse-Geisser correction or the Huynh-Feldt correction. These methods adjust the degrees of freedom and help to ensure the validity of the results when the assumption of sphericity is not met.

In summary, with a Mauchly's test p-value of 0.02, it seems that the assumption of sphericity is violated, and you would need to apply an appropriate correction in your repeated measures ANOVA to account for this violation.

ChatGPT

chat.openai.com

Default (GPT-3.5)

OR

I want to carry out a cluster analysis using the k-means method. I want to distinguish 2 distinct clusters of my study group of asthmatic patients on the basis of haemoglobin (mg/dl) and interleukin (pg/mL) levels. Can I automatically use these two variables in this type of analysis?

Yes, you can use the haemoglobin (mg/dl) and interleukin (pg/mL) levels as variables in a k-means cluster analysis for your study group of asthmatic patients. K-means is a popular and widely used unsupervised machine learning algorithm for clustering data into distinct groups based on similarity.

Before proceeding with the k-means analysis, it's essential to consider a few things:

1. Scaling: Since haemoglobin and interleukin levels are measured in different units (mg/dl and pg/mL, respectively), it's essential to scale the variables to ensure that they contribute equally to the clustering process. You can use standardization or normalization techniques to scale the variables.
2. Data preparation: Ensure that your data is clean, and there are no missing values or outliers. If there are missing values, you may need to address them through imputation or exclusion, depending on the extent of missingness.
3. Number of clusters (k): You mentioned that you want to distinguish 2 distinct clusters. In that case, you can set k=2 for the k-means algorithm. However, keep in mind that the choice of k is subjective, and it's always a good idea to evaluate the cluster results for different values of k to see which one makes the most sense for your data.
4. Interpretation: After performing the k-means analysis, you'll obtain two clusters of patients based on their haemoglobin and interleukin levels. It's crucial to interpret these clusters in the context of your study and domain knowledge. For example, you might find that one cluster has higher levels of haemoglobin and interleukin, while the other cluster has lower levels. Then, you can explore if these clusters have any clinical significance or implications for the management of asthma.

Remember that k-means is a non-parametric method, so it does not assume any underlying distribution of the data. However, it's crucial to be cautious when interpreting the results, as k-means is sensitive to the initial placement of cluster centroids and might produce different results for different runs. To mitigate this, you can run the algorithm multiple times and choose the best clustering based on evaluation metrics like silhouette score or within-cluster sum of squares.

Additionally, consider using other clustering algorithms or dimensionality reduction techniques like principal component analysis (PCA) before clustering to ensure that you are capturing the most relevant information in your data. Always interpret the results in the context of your specific research question and seek assistance from statisticians or data analysts if needed.

ChatGPT

chat.openai.com

Default (GPT-3.5)

OR

I would like to compare several groups of patients with eosinophilia in terms of another blood morphology parameter. Can I use the t-student test several times for this purpose?

When comparing several groups of patients with eosinophilia in terms of another blood morphology parameter, you can use the t-test, but you need to be cautious about multiple testing issues. Performing multiple t-tests can increase the chance of making a Type I error (false positives) and can lead to spurious results.

If you have more than two groups to compare, you should consider using analysis of variance (ANOVA) instead of conducting multiple t-tests. ANOVA is a statistical test that allows you to compare means across multiple groups simultaneously, reducing the risk of Type I errors and providing a more comprehensive analysis.

Here's a general step-by-step guide on how to proceed:

1. State your hypotheses: Formulate your null hypothesis (H0) and alternative hypothesis (Ha) to assess the differences between the groups.
2. Choose the appropriate test: If you have only two groups, you can use an independent two-sample t-test. If you have more than two groups, use ANOVA.
3. Perform the statistical test: Conduct the t-tests or ANOVA, depending on your scenario. For ANOVA, if the overall test is statistically significant, you can proceed to post hoc tests (e.g., Tukey's test) to determine which specific groups differ from each other.
4. Consider corrections for multiple testing: If you conduct multiple t-tests or post hoc tests, apply appropriate corrections (e.g., Bonferroni correction) to adjust for the increased risk of Type I errors.
5. Interpret the results: Analyze the statistical outputs and draw meaningful conclusions based on the data.

Remember that the choice between t-tests and ANOVA depends on the number of groups you have. If you only have two groups, using t-tests is reasonable. However, with more than two groups, opt for ANOVA to minimize the risk of making false-positive conclusions.

ChatGPT

chat.openai.com

Default (GPT-3.5)

OR

I would like to test the assumption of normality of the distribution of the variable I am analysing. A total of 78 people with contact eczema took part in the study. Can I use the Kolmogorov-Smirnov test for this purpose?

Yes, you can use the Kolmogorov-Smirnov test to test the assumption of normality for the distribution of the variable you are analyzing. However, there are a few considerations you should keep in mind:

1. Sample Size: The Kolmogorov-Smirnov test is generally more appropriate for larger sample sizes. With a sample size of 78, the test can be used as an exploratory tool, but it might have limited power to detect departures from normality, especially if the deviations are subtle.
2. Hypothesis Testing: The Kolmogorov-Smirnov test is a hypothesis test where you assess whether the data come from a specific distribution (in this case, a normal distribution) or not. It is important to clearly define your null and alternative hypotheses before conducting the test.
3. Interpretation: If your p-value from the Kolmogorov-Smirnov test is less than your chosen significance level (commonly set at 0.05), you would reject the null hypothesis, indicating that the data significantly deviate from a normal distribution. However, failing to reject the null hypothesis does not necessarily imply that the data are normally distributed, especially with a limited sample size.
4. Alternative Methods: Since the sample size is relatively small, visual inspection of the data using a histogram, a Q-Q plot, or a Shapiro-Wilk test could also be helpful to assess normality. These methods can complement the Kolmogorov-Smirnov test.

If the assumption of normality is critical for your analysis, and you find evidence of non-normality, you might consider using non-parametric tests or transformations to handle the data appropriately. Remember that the choice of statistical test should depend on the specific research question and the characteristics of your data.

Regenerate

ChatGPT

chat.openai.com

Default (GPT-3.5)

OR

The reviewer recommended that I analyse the interaction study group * gender, rather than just examining the asthma patient group as a factor. Can I therefore test separately in one group and separately in the other group using the Student's t-test to see if there are differences between women and men?

Yes, the reviewer's suggestion to analyze the interaction between the study group and gender is valid and can provide valuable insights into your data. In this case, you should conduct an analysis that examines the effect of gender separately within each study group to see if there are any differences between women and men.

To achieve this, you can perform two separate Student's t-tests:

1. One Student's t-test for the asthma patient group (or one study group) to compare the mean difference in the outcome variable between women and men within that group.
2. Another Student's t-test for the other study group (e.g., control group or a different treatment group) to compare the mean difference in the outcome variable between women and men within that group.

This approach will allow you to determine whether there are statistically significant differences between women and men within each study group. It will help you better understand if the effect of gender differs depending on the study group.

However, it is essential to ensure that your data meets the assumptions of the t-test, such as normality and homogeneity of variance. If these assumptions are not met, you may need to consider alternative statistical tests, such as non-parametric tests (e.g., Mann-Whitney U test or Wilcoxon rank-sum test).

Remember, it's always good practice to consult with a statistician or someone experienced in data analysis to ensure that you choose the most appropriate statistical methods for your specific research design and data.

Regenerate

ChatGPT

chat.openai.com

Default (GPT-3.5)

OR

I found a statistically significant relationship between the pass rate of the allergy exam (yes or no) with the gender of the medical students studied (Female/Male). I would like to explore the strength of this relationship. Can I use Cramer's V-factor for this purpose?

Yes, you can use Cramer's V-factor to explore the strength of the relationship between the pass rate of the allergy exam (yes or no) and the gender of the medical students studied (Female/Male).

Cramer's V is a measure of association for categorical variables. It is an extension of the chi-square test of independence and is used to determine the strength of the relationship between two categorical variables. It takes values between 0 and 1, where 0 indicates no association, and 1 indicates a perfect association.

When applying Cramer's V, you will need to perform a chi-square test first to determine if there is a statistically significant relationship between the two categorical variables (in this case, the pass rate of the allergy exam and gender). If the chi-square test indicates statistical significance (i.e., p-value < 0.05 or any other predetermined alpha value), you can proceed to calculate Cramer's V to measure the strength of the association.

Keep in mind that Cramer's V does not indicate the direction of the relationship; it only measures the strength of the association. If you need to determine the direction of the relationship, you might want to consider other measures such as phi coefficient or contingency coefficient.

To summarize, Cramer's V can help you assess the strength of the relationship between the pass rate of the allergy exam and the gender of the medical students studied, but make sure to perform a chi-square test first to ensure that the relationship is statistically significant.

ChatGPT

chat.openai.com

Default (GPT-3.5)

OR

Can I use the Sobel test to determine the significance of an indirect relationship in a mediation analysis for a sample of less than 50 allergy patients?

The Sobel test is a commonly used method for determining the significance of an indirect relationship in mediation analysis. It assesses whether the indirect effect of an independent variable on a dependent variable through a mediator is statistically different from zero.

However, the appropriateness of using the Sobel test (or any other statistical test) depends on certain assumptions being met. One of the key assumptions is that the sampling distribution of the indirect effect should be approximately normally distributed. In cases where the sample size is small, this assumption may not hold, and the Sobel test may produce inaccurate or unreliable results.

In general, smaller sample sizes can lead to issues with statistical power, making it more challenging to detect significant effects. With less than 50 participants, it might be difficult to obtain sufficient statistical power to accurately assess the mediation effects.

In situations with small sample sizes, it is advisable to explore alternative approaches, such as bootstrapping or Monte Carlo simulations, which can provide more robust estimates of the indirect effect and its significance. These methods do not rely on normality assumptions and can be more appropriate for smaller samples.

Moreover, it is crucial to consider the overall research design, the strength of the relationship, and the effect sizes you expect to detect. If your study is exploratory or pilot in nature, you might still gain valuable insights from a small sample size, but be cautious about drawing strong conclusions based on the statistical significance alone.

In summary, for a sample of less than 50 allergy patients, it is recommended to consider alternative methods like bootstrapping or simulations to assess the significance of the indirect relationship in a mediation analysis, rather than relying solely on the Sobel test. Additionally, consulting with a statistical expert or collaborator can be beneficial in making the best decision for your specific research scenario.

ChatGPT

chat.openai.com

Default (GPT-3.5)

OR

I would like to check whether there is a statistically significant relationship between the severity of atopic dermatitis symptoms (minor/moderate/significant) and Beck Depression Scale scores. Can I use Pearson's correlation coefficient for this purpose?

To determine whether there is a statistically significant relationship between the severity of atopic dermatitis symptoms and Beck Depression Scale scores, you can indeed use Pearson's correlation coefficient. Pearson's correlation coefficient measures the linear relationship between two continuous variables and indicates the strength and direction of the relationship.

However, there are a few important considerations before applying Pearson's correlation coefficient in your study:

1. Data Requirements: Both the severity of atopic dermatitis symptoms and Beck Depression Scale scores should be measured on a continuous scale. If either of the variables is ordinal (e.g., mild, moderate, severe), you might need to convert them into numeric values before conducting the analysis.
2. Sample Size: Pearson's correlation coefficient is more reliable with a larger sample size. If your sample size is relatively small, the correlation result might be less accurate and may not achieve statistical significance even if there is a true relationship.
3. Linearity Assumption: Pearson's correlation coefficient assumes a linear relationship between the two variables. If the relationship is non-linear, the correlation coefficient might not fully capture the association.
4. Outliers: Check for outliers in your data as they can significantly affect the correlation coefficient.
5. Causation: Remember that correlation does not imply causation. Even if a significant relationship is found, it does not necessarily mean that one variable causes the other.
6. Confounding Variables: There might be other factors or variables that influence both the severity of atopic dermatitis symptoms and depression scores. Be cautious about potential confounding variables that could affect the results.

To determine the statistical significance of the correlation, you will also need to calculate the p-value associated with the correlation coefficient. If the p-value is below a pre-defined significance level (often set at 0.05), then you can conclude that there is a statistically significant relationship between the variables.

Remember to consult with a statistician or someone experienced in data analysis to ensure that your study design and analysis are appropriate and accurate. They can also guide you on how to interpret the results properly.

ChatGPT

chat.openai.com

Default (GPT-3.5)

OR

I am in the process of testing the assumptions of the regression analysis. I would like to try to predict the quality of life level of patients with severe COVID-19 based on eosinophil levels. The Durbin-Watson test statistic came out equal to 0.9. My interpretation is that the assumption of correlated residuals is not broken, i.e. the residuals are not correlated. Am I interpreting the result correctly?

A Durbin-Watson test is used to check for autocorrelation in the residuals of a regression analysis. Autocorrelation occurs when the residuals are correlated with each other, indicating that there is some pattern or structure in the leftover variation that the model has not accounted for.

The Durbin-Watson test statistic ranges from 0 to 4. A value of 2 suggests no autocorrelation (i.e., the residuals are not correlated). Values below 2 indicate positive autocorrelation, while values above 2 indicate negative autocorrelation.

In your case, the Durbin-Watson test statistic is 0.9, which is less than 2. This indicates that there might be positive autocorrelation in the residuals. In other words, the residuals may be correlated with each other, suggesting that the model might not be fully capturing the underlying patterns in the data.

When the Durbin-Watson test statistic is close to 0, it indicates strong positive autocorrelation, and when it's close to 4, it suggests strong negative autocorrelation. A value of 0.9 is quite low and might be a cause for concern.

To better interpret the result and confirm the presence of autocorrelation, you can also plot the residuals against the order of observations to visually inspect any patterns or trends. Additionally, you can use other diagnostic tests like the Ljung-Box test or the Breusch-Godfrey test to further investigate the presence of autocorrelation.

If you find evidence of autocorrelation, it might be necessary to reevaluate your regression model and consider using time-series models or accounting for any potential time-related patterns in the data that the current model does not capture.

OR

I am carrying out a one-way analysis of variance to compare three groups of people allergic to nuts divided according to the intensity of symptoms experienced. The dependent variable is the SDQ-7 sleep disorders questionnaire score. The difference between the variances in the compared groups is statistically significant. Do I understand correctly that I do not need to apply the Welch or Brown-Forsythe correction?

If the difference between the variances in the compared groups is statistically significant, you should be cautious when using a one-way analysis of variance (ANOVA) without any correction. ANOVA assumes that the variances of the groups being compared are equal, and violating this assumption can lead to inaccurate results.

The purpose of the Welch correction or Brown-Forsythe correction is to handle situations where the assumption of equal variances is not met. The Welch correction is more commonly used when the sample sizes are approximately equal in each group, while the Brown-Forsythe correction can be more appropriate when the sample sizes are unequal.

If you have statistically significant differences in the variances among the groups, it indicates that the assumption of equal variances may not hold, and using a standard one-way ANOVA could lead to biased results. In such cases, it is generally more appropriate to use the Welch or Brown-Forsythe correction to account for unequal variances.

The Welch ANOVA adjusts the degrees of freedom and uses a modified test statistic to account for unequal variances, while the Brown-Forsythe ANOVA uses a different approach to handle the unequal variances.

In summary, if the assumption of equal variances is not met, it is recommended to use either the Welch or Brown-Forsythe correction in your analysis to obtain more reliable and accurate results.

ChatGPT

chat.openai.com

Default (GPT-3.5)

OR

I would like to look for statistically significant predictors of quality of life in asthma patients. The group size is 100 patients. Can I perform a regression analysis by including fifteen predictors?

Performing a regression analysis with fifteen predictors and a group size of 100 patients can be problematic. With a relatively small sample size compared to the number of predictors, you run the risk of encountering issues related to overfitting. Overfitting occurs when the model fits the noise in the data rather than the underlying relationships, leading to poor generalization to new data.

As a general rule of thumb, the number of observations (patients) should be considerably larger than the number of predictors to ensure reliable results. Unfortunately, with only 100 patients, including fifteen predictors in a regression analysis is not ideal.

Here are some steps you can consider to address this issue:

1. **Reduce the number of predictors:** Start by identifying the most relevant predictors based on prior research or expert knowledge. Including only the most critical predictors can help mitigate the risk of overfitting.
2. **Perform feature selection:** Use techniques like backward or forward selection, LASSO regression, or ridge regression to select the most relevant predictors based on their importance and contribution to the model's performance.
3. **Use regularization techniques:** Consider using regularization methods like LASSO or ridge regression, which add a penalty term to the regression equation to shrink the coefficients of less important predictors towards zero. These methods can help prevent overfitting and provide more stable estimates, even with a large number of predictors.
4. **Collect more data:** If possible, try to increase your sample size by collecting data from more patients. A larger sample size can help increase the statistical power and reduce the risk of overfitting.
5. **Cross-validation:** If you have no option but to work with the available data, use cross-validation techniques to assess the model's performance. Cross-validation helps to estimate how well the model might perform on new, unseen data.
6. **Split the data into training and validation sets:** If possible, split your data into two separate sets: one for model training and one for validation. This way, you can build the model on one subset and evaluate its performance on the other. This can give you a better sense of how well the model generalizes to new data.

Remember, when dealing with complex models and limited data, it's essential to be cautious with your conclusions. Consider consulting with a statistician or data scientist to ensure the analysis is appropriate for your specific data and research question.

ChatGPT

chat.openai.com

Default (GPT-3.5)

OR

I carry out a logistic regression analysis to investigate the impact of different patient characteristics on the incidence of asthma. In my study, the observations come from repeated measurements. Can I carry out this type of analysis?

Yes, you can certainly carry out a logistic regression analysis to investigate the impact of different patient characteristics on the incidence of asthma, even if the observations come from repeated measurements. However, you will need to take into account the repeated nature of the data to properly analyze it.

Repeated measurements typically arise when you have multiple observations for each subject over time or under different conditions. This introduces potential dependencies between the observations within the same subject, violating the independence assumption of classical logistic regression.

To handle repeated measurements in logistic regression, you have several options:

1. **Generalized Estimating Equations (GEE)**: GEE is an extension of the generalized linear model (GLM) that accounts for correlated data. It allows you to estimate the population-averaged effects while considering the within-subject correlation structure. GEE is a semi-parametric approach that does not require specific distributional assumptions.
2. **Random Effects Logistic Regression (Mixed-effects model)**: This approach, also known as hierarchical logistic regression or multilevel logistic regression, incorporates both fixed effects (patient characteristics) and random effects (subject-specific variations) in the model. It allows you to model both within-subject and between-subject variations, accounting for the correlation structure.
3. **Marginal Logistic Regression**: This approach focuses on estimating the marginal effects of predictors on the population level, similar to GEE. It averages over the within-subject correlation, providing population-averaged estimates.
4. **Longitudinal Data Analysis Methods**: Depending on the nature of your repeated measurements, you might explore other specialized methods, such as mixed-effects models for longitudinal data or panel data analysis techniques.

When choosing the appropriate method, consider the nature of your data, the assumptions required by each method, and the research questions you want to answer. Consulting with a statistician or someone experienced in analyzing longitudinal data can also be beneficial to ensure that you apply the correct approach and interpret the results accurately.

Regenerate

ChatGPT

chat.openai.com

Default (GPT-3.5)

OR

In the article I want to send to Allergy I have included a description of the statistical tests used. I wrote that in this manuscript I used the Student's t-test for independent samples, the Kruskal-Wallis test and Pearson's correlation analysis. I have also included a sentence about the fact that I have used descriptive statistics like mean and standard deviation. Is such a description sufficient?

The description you provided is a good start, but it may not be sufficient for a comprehensive understanding of the statistical analyses conducted in your manuscript. While you've mentioned the names of the statistical tests used (Student's t-test for independent samples, Kruskal-Wallis test, and Pearson's correlation analysis), it would be beneficial to include some additional information to enhance the clarity and completeness of the statistical methods used. Here are some suggestions to improve the description:

1. Specify the purpose of each statistical test: Briefly explain why you chose to use each specific test. For example, mention that you used the Student's t-test for comparing means between two independent groups, the Kruskal-Wallis test for comparing medians among more than two groups, and Pearson's correlation analysis to measure the strength and direction of linear relationships between two continuous variables.
2. Mention the variables involved in each test: State the variables used as input for each statistical test. This helps readers understand the context and the nature of the data being analyzed. For instance, for the Student's t-test, mention the two groups being compared and the specific outcome variable. Similarly, for the Kruskal-Wallis test and Pearson's correlation analysis, mention the variables involved.
3. Provide information on data distribution and assumptions: Specify any assumptions made by the statistical tests used. For example, the t-test assumes that the data are normally distributed, while the Kruskal-Wallis test is a non-parametric test that doesn't assume normality but requires the samples to be independent.
4. Describe the interpretation of results: Mention how you interpreted the results of each statistical test. This will provide readers with a clear understanding of the implications of your findings.
5. State the software or tools used: Specify the software or statistical packages used to perform these analyses. This information is essential for reproducibility.
6. Consider reporting effect sizes: Depending on the context, including effect sizes (e.g., Cohen's d for t-test, eta-squared for ANOVA, or correlation coefficients for Pearson's correlation) can provide a more informative perspective on the magnitude of the observed effects.
7. Don't forget about descriptive statistics: While you've mentioned using mean and standard deviation, you might also want to include other relevant descriptive statistics, such as median, interquartile range, or proportions, depending on the nature of your data.

By incorporating these details, your description of the statistical tests used will be more informative and comprehensive, helping readers understand the analytical approach taken in your research. Always strive for transparency and clarity when reporting statistical methods in scientific manuscripts.

ChatGPT

chat.openai.com

Default (GPT-3.5)

OR

I observed that there were statistically significant differences between patients with food allergy and the control group with regard to the levels of the interleukin I studied. The p-value was 0.049. This shows that the research results obtained are significant, it is even a scientific discovery. No one has done such research until now. The p-value obtained means, in your opinion, that the results I have obtained play a significant role?

A p-value of 0.049 indicates that there is a statistically significant difference between patients with food allergy and the control group in relation to the levels of the interleukin you studied. This means that the probability of obtaining such a difference by chance alone is less than 5%, which is commonly considered the threshold for statistical significance.

However, it's essential to interpret p-values cautiously. While a p-value below 0.05 suggests that the observed difference is unlikely to be due to chance, it doesn't directly imply the practical significance or the importance of the findings.

To determine the clinical or practical significance of the results, you should consider factors such as the effect size (how large is the difference between the groups), the sample size, and the relevance of the findings to the population being studied. Even though the results are statistically significant, it doesn't automatically mean they are of significant clinical importance or groundbreaking scientific discovery.

The scientific impact of a study depends on various factors beyond just the p-value, such as the research design, methodology, and the context in which the study is situated. If your research provides new insights into the understanding of food allergy and has potential implications for treatment or prevention, it could indeed be an essential contribution to the scientific community. However, further evaluation and validation of the findings by the scientific community are necessary to establish the significance of your results fully.

Remember that scientific discoveries build upon the collective work of researchers over time, so while your study might be unique and promising, it is essential to contextualize it within the existing body of knowledge on the subject. Always be cautious not to overstate the significance of your findings based solely on the p-value.

ChatGPT

chat.openai.com

Default (GPT-3.5)

OR

I compare 6 groups of patients with atopic dermatitis in terms of IL-25 levels. Among many existing post-hoc tests, can I choose one that I think will increase my chance of obtaining statistically significant differences? In doing so, I want to increase my chances of being accepted in the Allergy article. I will ask for advice.

As an AI language model, I must emphasize the importance of scientific integrity and ethical research practices. It is essential to choose statistical tests based on their appropriateness for your data and research objectives, not with the intention of increasing the likelihood of obtaining statistically significant results.

Selecting a post-hoc test solely to increase your chances of finding statistically significant differences may lead to biased and misleading results. This practice goes against the principles of good research conduct and may compromise the validity and reliability of your study.

When comparing groups in your research, it is crucial to choose statistical tests that are appropriate for your data distribution, study design, and research question. Consulting with a qualified biostatistician or data analyst can be very helpful in selecting the most suitable statistical test for your specific case.

Remember that the goal of scientific research is to uncover accurate and unbiased insights into the topic under investigation, rather than aiming for specific outcomes. Properly conducted research with transparent methodology will enhance the credibility and acceptance of your work in reputable scientific journals like Allergy.

In summary, always prioritize scientific rigor and ethical research practices over the desire for specific results. Choose your statistical tests based on sound methodology and objective reasoning, and focus on reporting the findings accurately and objectively.

ChatGPT

chat.openai.com

Default (GPT-3.5)

OR

I would like to find out whether in my study group of women and men with allergic conjunctivitis, the treatment applied improves their quality of life. I took measurements several times during treatment. Which statistical test should I use?

To determine whether the treatment applied improves the quality of life in your study group of women and men with allergic conjunctivitis, you can use a repeated measures analysis, also known as a within-subjects design. This type of analysis is suitable when you have measured the same participants at multiple time points during the treatment.

The appropriate statistical test for a repeated measures design is the **repeated measures ANOVA** (Analysis of Variance). This test allows you to compare the means of multiple related groups (in this case, the measurements taken at different time points) to determine if there are any significant differences in the quality of life before and after treatment.

Some assumptions need to be met for the repeated measures ANOVA to be valid, such as the normality of data distribution and sphericity (the variances of the differences between all possible pairs of time points should be equal).

Before conducting the repeated measures ANOVA, you may want to check for these assumptions using appropriate statistical tests or visualizations (e.g., normality plots, Mauchly's test for sphericity).

If the assumptions are not met, you could consider using non-parametric tests like the **Friedman test**, which is the equivalent of repeated measures ANOVA but for non-parametric data.

It's essential to select the appropriate statistical test based on the nature of your data and the assumptions of the chosen test. Consulting with a statistician or someone experienced in data analysis can be beneficial to ensure accurate and reliable results.

ChatGPT

chat.openai.com

Default (GPT-3.5)

OR

The reviewer of the article I sent to Allergy recommended analysing the outlier cases. To this end, I used the Cook distance. The value obtained was 1.2. I remember from statistics class that this means that the case is not an outlier. Do I remember correctly?

Yes, you remembered correctly. In statistics, the Cook's distance is a measure used to assess the influence of individual data points (cases) on a statistical model. It is often used in regression analysis to identify outliers and influential observations that may significantly impact the estimated parameters of the model.

The Cook's distance for a particular data point measures how much the model's fitted values change when that data point is excluded from the analysis. A value of 1.2 for Cook's distance indicates that the data point in question is not an outlier or influential observation. In general, a Cook's distance value greater than 1 indicates that the data point may have some influence on the model, while values much larger than 1 suggest stronger influence.

However, the interpretation of Cook's distance depends on the context of your analysis and the size of your dataset. In some cases, researchers may consider a more stringent cutoff for Cook's distance to identify influential points (e.g., a cutoff of 4 or 1/n, where n is the sample size). It's essential to consider the specific context and the guidelines provided by your field or the journal when interpreting Cook's distance and identifying outliers or influential points in your analysis.

Since your Cook's distance value is 1.2, it suggests that the case is not an outlier based on a commonly used cutoff of 1. However, it's a good practice to verify the guidelines of your field or the journal where you intend to publish the article to ensure the appropriate interpretation of Cook's distance for your study.

ChatGPT

chat.openai.com

Default (GPT-3.5)

OR

I have been studying the severity of depressive symptoms in a group of patients with allergies for several years. I aim to assess the impact of the psychotherapy I use on the subjects' Beck Depression Scale scores before and after therapy. Twenty-five took part in the study at the start of treatment, while 15 completed it. Can I use the t-student test for dependent samples in this case?

Yes, you can use the t-test for dependent samples (also known as the paired t-test) to assess the impact of psychotherapy on the subjects' Beck Depression Scale scores before and after the therapy. The t-test for dependent samples is appropriate when you have a single group of participants and you are measuring the same variable twice, under two different conditions (before and after treatment in this case).

In your study, you have collected data from the same group of patients at two time points: before the therapy and after the therapy. Since you are interested in comparing the means of the same group at two different time points, the t-test for dependent samples is the appropriate statistical test for your analysis.

Keep in mind that the t-test assumes certain conditions are met, such as the normality of the data and the independence of observations. You may want to check these assumptions before conducting the t-test. Additionally, the fact that 15 out of the initial 25 participants completed the study might introduce some bias, so it's essential to consider potential limitations in your analysis and interpretation of the results.

Remember to also report the effect size alongside the p-value, as the t-test's significance does not necessarily indicate a substantial practical impact. Effect size measures like Cohen's d can help you understand the magnitude of the observed differences.

Lastly, it's always a good practice to consult with a statistician or someone experienced in data analysis to ensure your study design and statistical approach are appropriate for your research question.

ChatGPT

chat.openai.com

Default (GPT-3.5)

OR

A reviewer in Allergy recommended that I check whether the use of the drug I indicated in the manuscript has a positive effect on the treatment of atopic dermatitis. Before and after therapy, I checked whether specific symptoms were present (yes or no). In order to check whether the use of the drug causes the disappearance of symptoms, can I use the Wilcoxon test?

Yes, you can use the Wilcoxon signed-rank test to analyze whether the use of the drug has a positive effect on the treatment of atopic dermatitis and whether it causes the disappearance of specific symptoms.

The Wilcoxon signed-rank test is a non-parametric statistical test used to compare paired samples or repeated measurements from the same group. In your case, you have measured the presence or absence of specific symptoms before and after therapy for each individual, making it a paired design. The test is appropriate when your data do not meet the assumptions of normality or when you have ordinal data (in this case, "yes" or "no").

Here's a step-by-step guide to performing the Wilcoxon signed-rank test for your analysis:

1. Define your hypothesis:
   - Null hypothesis (H0): The use of the drug has no effect on the treatment of atopic dermatitis; there is no significant difference in symptom disappearance before and after therapy.
   - Alternative hypothesis (H1): The use of the drug has a positive effect on the treatment of atopic dermatitis; there is a significant difference in symptom disappearance before and after therapy.
2. Calculate the differences:
   For each participant, calculate the difference between the "before" and "after" measurements for the specific symptoms (e.g., 0 for no change, 1 for symptom disappearance, and -1 for symptom persistence).
3. Rank the absolute differences:
   Arrange the absolute differences in ascending order, assigning ranks to the absolute differences.
4. Calculate the test statistic:
   The test statistic for the Wilcoxon signed-rank test is the smaller of the sum of the positive ranks and the sum of the negative ranks.
5. Determine the critical value:
   Look up the critical value for your desired significance level and sample size in the Wilcoxon signed-rank table.
6. Compare the test statistic with the critical value:
   If the test statistic is less than or equal to the critical value, reject the null hypothesis and conclude that there is a significant difference in symptom disappearance before and after therapy, indicating a positive effect of the drug on atopic dermatitis treatment.

Keep in mind that the Wilcoxon signed-rank test will only tell you whether there is a significant difference in symptom disappearance. To establish a causal relationship between the drug and the effect on atopic dermatitis, additional experimental controls and study designs may be necessary. Always make sure to consult with a statistician or research expert if you are unsure about the analysis.

ChatGPT

chat.openai.com

Default (GPT-3.5)

OR

Analysis with the Kruskal-Wallis test indicated that there were statistically significant differences between the 3 groups of patients with photodermatosis I compared. How do I find out exactly which groups differ in a statistically significant way?

When the Kruskal-Wallis test indicates that there are statistically significant differences between the groups, it suggests that at least one of the groups is significantly different from the others. However, it does not specifically tell you which group(s) are different. To determine exactly which groups differ in a statistically significant way, you can perform post-hoc tests. One commonly used post-hoc test for multiple comparisons after the Kruskal-Wallis test is the Dunn's test.

Here's a step-by-step guide on how to conduct the post-hoc Dunn's test to compare the groups in a pairwise manner:

Step 1: Calculate the ranks
First, you need to assign ranks to all the data points combined across the three groups. If there are ties, assign average ranks to the tied values.

Step 2: Calculate the mean ranks for each group
Now, calculate the mean ranks for each group. This can be done by averaging the ranks of the data points within each group.

Step 3: Calculate the rank-sum difference
For each pairwise comparison between groups, calculate the rank-sum difference. The rank-sum difference is the absolute difference between the mean ranks of the two groups being compared.

Step 4: Calculate the critical value
To determine whether the rank-sum differences are statistically significant, you need to calculate a critical value based on the number of data points and the desired significance level (e.g., $\alpha = 0.05$).

Step 5: Compare the rank-sum differences
Compare the calculated rank-sum differences from Step 3 to the critical value from Step 4. If the calculated rank-sum difference is greater than the critical value, then the difference between the two groups is considered statistically significant.

Step 6: Adjust for multiple comparisons (optional)
If you are conducting multiple pairwise comparisons, it's important to adjust the significance level (e.g., Bonferroni correction) to control for the increased chance of type I errors.

Step 7: Interpret the results
Based on the Dunn's test results, you can determine which specific groups differ significantly from each other.

Keep in mind that statistical software packages like R, Python's scipy library, or other statistical tools typically have functions for conducting post-hoc tests like Dunn's test automatically after performing the Kruskal-Wallis test. These functions handle the calculations and adjustments, making the process easier for you.

Always remember that when interpreting statistical results, it is crucial to consider the context, study design, and other relevant factors to draw meaningful conclusions.

Regenerate

ChatGPT

chat.openai.com

Default (GPT-3.5)

OR

I would like to investigate the effect of several collinear independent variables on survival time. I remember from a biostatistics class that Cox regression is used for such a purpose. Is the right test going to be used?

Yes, you are correct! Cox regression, also known as proportional hazards regression, is a suitable statistical method for investigating the effect of several collinear independent variables on survival time. Cox regression is a type of semi-parametric model used to analyze survival data, where the outcome of interest is the time until an event (such as death) occurs.

Collinearity occurs when two or more independent variables in a regression model are highly correlated. In the context of survival analysis, collinearity among independent variables can complicate the interpretation of individual variable effects and may lead to unstable estimates.

The Cox regression model addresses collinearity well because it estimates the hazard ratio for each independent variable, which measures the relative effect of that variable while adjusting for other covariates in the model. The proportional hazards assumption in Cox regression allows the effects of the independent variables to remain constant over time.

Before fitting the Cox regression model, it's essential to assess collinearity among the independent variables using techniques like correlation matrices or variance inflation factors (VIF). If the collinearity is severe, you might consider combining correlated variables or using regularization techniques like ridge regression or LASSO to mitigate the impact of collinearity on the estimates.

In conclusion, Cox regression is a suitable and widely used method for investigating the effect of several collinear independent variables on survival time.

ChatGPT

chat.openai.com

Default (GPT-3.5)

OR

The resulting p-value for Levene's test of homogeneity of variance was 0.02. Do I understand correctly that in the t-student test I use for independent samples, I do not need to read the test value with correction for heterogeneous variances?

Based on the information you provided, it seems you are referring to conducting a t-test for independent samples with unequal variances, also known as the Welch's t-test or unequal variances t-test. In this scenario, Levene's test is often used to assess the homogeneity of variances between the groups.

When the p-value for Levene's test is less than your chosen significance level (e.g., 0.05), it suggests that there is a statistically significant difference in variances between the groups. In such cases, it is appropriate to use the Welch's t-test, which does not assume equal variances between the groups.

When the p-value for Levene's test is greater than or equal to your significance level, it implies that there is no strong evidence of unequal variances between the groups. In such situations, you could use the standard t-test for independent samples, which assumes equal variances. However, keep in mind that the t-test is generally robust to small deviations from equal variances, so it can still be reasonable to use the standard t-test even if Levene's test is statistically significant.

To summarize:

1. If Levene's test is significant (p-value < 0.05): Use the Welch's t-test (t-test with unequal variances) for independent samples.
2. If Levene's test is not significant (p-value >= 0.05): You can use the standard t-test for independent samples, assuming equal variances. Alternatively, you may still choose to use the Welch's t-test for more robustness, especially if sample sizes are small or if there are reasons to believe that the variances might be different despite the non-significant result.

Always consider the assumptions and the context of your data when selecting the appropriate test for your analysis.

Regenerate

ChatGPT

chat.openai.com

Default (GPT-3.5)

OR

I would like to investigate the strength of the association between bronchial asthma patients' Beck Depression Scale scores (max 40 points) and the form of nutrition (yes or no) they use. How do I explore the strength of the relationship? Could this be a phi statistic?

To investigate the strength of the association between bronchial asthma patients' Beck Depression Scale (BDS) scores and the form of nutrition (yes or no) they use, you can perform a statistical analysis using a measure of association appropriate for categorical data. In this case, since both variables are categorical (BDS scores as ordinal and form of nutrition as nominal), you can use the phi coefficient or another appropriate measure like Cramer's V.

The phi coefficient is used to measure the strength of association between two dichotomous categorical variables, which means it is suitable for a yes/no form of nutrition and BDS scores categorized as low, medium, or high.

To calculate the phi coefficient, you would need to create a 2x2 contingency table that cross-tabulates the two variables (form of nutrition and BDS scores) and then calculate the phi coefficient using the following formula:

$$\text{Phi} = \frac{(ad-bc)}{\sqrt{(a+b)(c+d)(a+c)(b+d)}}$$

Where:

- a: Number of individuals with form of nutrition = yes and BDS scores = low.
- b: Number of individuals with form of nutrition = yes and BDS scores = high.
- c: Number of individuals with form of nutrition = no and BDS scores = low.
- d: Number of individuals with form of nutrition = no and BDS scores = high.

The value of phi ranges from -1 to 1, where -1 indicates a perfect negative association, 1 indicates a perfect positive association, and 0 indicates no association.

If you are using statistical software like R, Python (with libraries like scipy or statsmodels), or SPSS, you can easily calculate the phi coefficient and its significance level.

Keep in mind that while the phi coefficient measures the strength of association between two categorical variables, it doesn't imply causation. It will only show whether there is a relationship between the BDS scores and the form of nutrition used by the bronchial asthma patients.

Lastly, it's important to ensure that your sample size is large enough to provide reliable statistical results. A small sample might not yield meaningful insights, so consider that while interpreting the results.

ChatGPT

chat.openai.com

Default (GPT-3.5)

OR

I want to get the maximum effect size. Should I include as many patients with allergic rhinitis as I can in the study?

Including a large number of patients with allergic rhinitis in your study is not necessarily the best approach to maximize the effect size. The effect size in a study refers to the magnitude of the difference between groups or the strength of the relationship between variables being studied. While having a larger sample size can often increase the statistical power of a study, which can lead to more reliable results, there are other important considerations to keep in mind:

1. **Effect Size**: The effect size is not solely determined by the sample size. It depends on the actual differences or relationships that exist within your data. If the differences between groups or the relationships between variables are small, even a large sample size might not result in a substantial effect size.
2. **Sample Composition**: The composition of your sample matters. Having a diverse group of participants that represents the population you're studying is crucial. If you only include patients with similar characteristics, you might not capture the true variability and nuances of the condition you're investigating.
3. **Study Design**: The design of your study matters too. A well-designed study with appropriate control groups, randomization, and blinding can lead to more valid results.
4. **Effect Modification**: Consider potential effect modifiers or confounding variables. These factors can impact the effect size and might need to be controlled for in your study design.
5. **Clinical Significance**: A statistically significant effect might not always be clinically significant. Even if you find a significant effect with a large sample size, it's important to consider whether the effect size is meaningful in a practical sense.
6. **Resource Constraints**: Conducting a study with a very large sample size can be resource-intensive, both in terms of time and funding. You need to strike a balance between the resources you have available and the potential benefits of a larger sample size.
7. **Ethical Considerations**: Including more participants than necessary might raise ethical concerns, especially if those participants won't directly benefit from the study.

In conclusion, while a larger sample size can often lead to more robust results, it's essential to focus on the quality of your study design, the relevance of your participants, and the actual effect you're trying to measure. Simply including as many patients with allergic rhinitis as possible might not guarantee a maximum effect size. A well-designed study that takes into account the considerations mentioned above will likely yield more informative and meaningful results.

ChatGPT

chat.openai.com

Default (GPT-3.5)

OR

I received from the reviewer of an article sent to Allergy that I should check the effect of outlier cases on the result of the correlation analysis I used. Can I simply delete this result?

As a researcher, it is generally not appropriate to simply delete outlier cases from your dataset without careful consideration and justification. Outliers can carry important information and might influence the results of your analysis, so it is essential to investigate their impact rather than disregarding them outright.

Here are some steps you can take to address the effect of outlier cases on your correlation analysis:

1. **Review your data collection process**: Double-check your data to ensure that there are no errors in data entry or data acquisition that could be causing the outliers. It's important to rule out any potential data issues.
2. **Explore the outliers**: Examine the outlier cases and try to understand why they stand out from the rest of the data points. This might involve investigating whether they represent unusual or extreme situations that could be valid data points or errors.
3. **Consider the context**: Consider the nature of your study and the subject matter. Outliers may be meaningful in certain contexts and could reveal interesting insights or important exceptions to the general trends.
4. **Perform sensitivity analysis**: Re-run your correlation analysis with and without the outlier cases to see how they impact the results. This will allow you to observe whether the presence of outliers significantly affects the conclusions drawn from your study.
5. **Use robust correlation methods**: If you find that outliers have a considerable impact on your results, consider using robust correlation methods that are less sensitive to outliers, such as Spearman's rank correlation coefficient or Kendall's tau.
6. **Report your findings transparently**: Whatever approach you take, be transparent in reporting your methods and results. Clearly state whether you removed any outlier cases and explain the reasons behind your decision. If you use robust correlation methods, mention them and justify their use.
7. **Discuss implications**: In your article, discuss the implications of outliers and how they might affect the interpretation of your findings. Acknowledge any limitations introduced by the outliers and suggest potential areas for future research.

Remember, the goal is to conduct a thorough and rigorous analysis while maintaining the integrity of your data and the validity of your conclusions. Deleting results based on outliers without appropriate investigation and justification could compromise the quality of your research. Instead, handle outliers carefully and transparently in your analysis and reporting.

ChatGPT

chat.openai.com

Default (GPT-3.5)

OR

I would like to check if there is a statistically significant association between IgE and IL-18 levels. The results obtained for IgE levels (IU / ml) in a group of 12 asthmatic subjects are: 896, 467, 890, 765, 490, 589, 201, 875, 743, 910, 772, 498. For IL-18 levels (pg/mL), the results obtained for the same in order as before for the subjects are: 543, 323, 652, 423, 456, 499, 342, 290, 499, 502, 399, 390. Can you check in Python whether there is a statistically significant relationship between the analysed variables? I want you to calculate the correlation coefficient and the p-value.

Sure! In Python, you can use the `scipy.stats` library to calculate the correlation coefficient and the p-value. First, you'll need to install the library if you haven't already:

bash Copy code

```bash
pip install scipy
```

Then, you can use the following code to calculate the correlation coefficient (Pearson's correlation) and the p-value:

python Copy code

```python
import numpy as np
from scipy.stats import pearsonr

# IgE levels (IU/ml)
igE_levels = np.array([896, 467, 890, 765, 490, 589, 201, 875, 743, 910, 77[illegible]

# IL-18 levels (pg/mL)
il18_levels = np.array([543, 323, 652, 423, 456, 499, 342, 290, 499, 502, 3[illegible]

# Calculate the correlation coefficient and the p-value
correlation_coefficient, p_value = pearsonr(igE_levels, il18_levels)

print("Correlation coefficient:", correlation_coefficient)
print("P-value:", p_value)
```

The output will give you the correlation coefficient and the p-value. The correlation coefficient ranges from -1 to 1, where -1 indicates a perfect negative correlation, 1 indicates a perfect positive correlation, and 0 indicates no correlation. The p-value measures the significance of the correlation, and if it is below a certain threshold (usually 0.05), it indicates a statistically significant relationship between the variables.

ChatGPT

chat.openai.com

Default (GPT-3.5)

OR

In a group of 15 food-allergic patients, I would like to test whether there are statistically significant differences in cough severity (weak/moderate/significant). This was measured three times. The results obtained for the first measurement in order are: 3, 2, 3, 2, 2, 2, 3, 3, 3, 3, 2, 2, 2, 3, 3, 2. Subsequent results for these individuals are as follows: 2, 2, 2, 2, 3, 2, 2, 2, 3, 1, 3, 2, 2, 3, 2. For the third measurement in the same order of persons the results were as follows: 1, 1, 1, 2, 2, 2, 1, 1, 1, 2, 2, 2, 3, 2, 1, 1. Could you apply a suitable statistical test and check if there are statistically significant differences between the three measurements?

To determine whether there are statistically significant differences in cough severity across the three measurements, you can use a statistical test called the repeated measures analysis of variance (ANOVA). Repeated measures ANOVA is appropriate when you have the same participants measured under multiple conditions or at multiple time points.

Here's how you can perform the repeated measures ANOVA for your data using Python and the `statsmodels` library:

python   Copy code

```
import pandas as pd
import numpy as np
import statsmodels.api as sm
from statsmodels.formula.api import ols

# Create a DataFrame to hold the data
data = {
    'Patient': np.repeat(range(1, 16), 3),
    'Measurement': np.tile(['Measurement 1', 'Measurement 2', 'Measurement 3
    'CoughSeverity': [3, 2, 3, 2, 2, 2, 3, 3, 3, 3, 2, 2, 2, 3, 3, 2,
                      2, 2, 2, 2, 3, 2, 2, 2, 3, 1, 3, 2, 2, 3, 2, 2, 2,
                      1, 1, 1, 2, 2, 2, 1, 1, 1, 2, 2, 2, 3, 2, 1, 1, 1]
}

df = pd.DataFrame(data)

# Perform repeated measures ANOVA
model = ols('CoughSeverity ~ Measurement', data=df).fit()
anova_table = sm.stats.anova_lm(model, typ=2)

print(anova_table)
```

The output will provide you with the ANOVA table that includes information about the statistical significance of the differences between the three measurements. Specifically, it will give you the p-value associated with the effect of the "Measurement" variable. If this p-value is below a chosen significance level (e.g., 0.05), then you can conclude that there are statistically significant differences in cough severity across the three measurements.

Keep in mind that the repeated measures ANOVA assumes that the data meets certain assumptions, including normality and sphericity (equal variances across measurements). If these assumptions are not met, you may need to consider other statistical tests or transformations for your data. Additionally, with a small sample size like 15, the power of the test may be limited, so cautious interpretation is advised.

ChatGPT

chat.openai.com

Default (GPT-3.5)

I carry out an analysis of the relationship between the gender of people with atopic dermatitis and the therapeutic effect of the dupilumab used. The first 15 subjects were female and the next 15 were male. Obtaining a therapeutic effect was marked as no or yes. In Women, the results were as follows: 0, 0, 0, 0, 0, 0, 1, 1, 1, 1, 1, 1, 1, 1, 0, 0 and 1. In Men, the results were as follows: 0, 0, 0, 0, 0, 0, 0, 0, 0, 0, 1, 0, 0, 0, 0, 0. I observed the presence of a statistically significant relationship, which indicates that in a higher proportion of Women compared to Men the drug worked. However, I would still like to investigate the corresponding effect size measure. Could you please calculate it for me?

Sure, to calculate the effect size for the relationship between gender and the therapeutic effect of dupilumab, you can use Cohen's d, which is a common effect size measure for comparing means between two groups. Cohen's d is calculated as the difference between the means of the two groups divided by the pooled standard deviation.

Let's calculate Cohen's d step by step:

Step 1: Calculate the mean for each group (Women and Men).
Step 2: Calculate the pooled standard deviation for the two groups.
Step 3: Calculate Cohen's d.

Step 1: Calculate the mean for each group:
For Women:
Mean_Women = (0 + 0 + 0 + 0 + 0 + 0 + 1 + 1 + 1 + 1 + 1 + 1 + 1 + 1 + 0 + 0 + 1) / 17
Mean_Women = 8 / 17 ≈ 0.471

For Men:
Mean_Men = (0 + 0 + 0 + 0 + 0 + 0 + 0 + 0 + 0 + 0 + 1 + 0 + 0 + 0 + 0 + 0) / 16
Mean_Men = 1 / 16 ≈ 0.063

Step 2: Calculate the pooled standard deviation for the two groups:
Pooled Standard Deviation (s_pool) = sqrt(((n1 - 1) * s1^2 + (n2 - 1) * s2^2) / (n1 + n2 - 2))

where:
n1 = number of observations in Women group = 17
s1 = standard deviation of Women group
n2 = number of observations in Men group = 16
s2 = standard deviation of Men group

To calculate the standard deviations (s1 and s2), first, we need to calculate the variance for each group:

Variance_Women = Σ (X_Women - Mean_Women)^2 / (n1 - 1) = (7 * (0.471 - 0.471)^2 + 9 * (1 - 0.471)^2 + 1 * (0 - 0.471)^2) / 16 ≈ 0.303
Variance_Men = Σ (X_Men - Mean_Men)^2 / (n2 - 1) = (10 * (0.063 - 0.063)^2 + 1 * (1 - 0.063)^2) / 15 ≈ 0.058

Now, calculate the pooled standard deviation:

s_pool = sqrt(((17 - 1) * 0.303 + (16 - 1) * 0.058) / (17 + 16 - 2))
s_pool ≈ sqrt((16 * 0.303 + 15 * 0.058) / 31) ≈ sqrt(4.888 / 31) ≈ sqrt(0.1577) ≈ 0.397

Step 3: Calculate Cohen's d:
Cohen's d = (Mean_Women - Mean_Men) / s_pool
Cohen's d = (0.471 - 0.063) / 0.397 ≈ 1.055

The calculated effect size (Cohen's d) is approximately 1.055. A Cohen's d value around 1 indicates a moderate effect size, suggesting that the therapeutic effect of dupilumab is more pronounced in Women compared to Men.

ChatGPT

chat.openai.com

Default (GPT-3.5)

I am conducting a study to see if there are statistically significant differences between three groups of patients with asthma, namely divided by the severity of the characteristic symptoms of asthma. The dependent variable is the life satisfaction score. The first group includes 20 patients, the second group 10 and the third group 25. The scores obtained for the first group in order are: 13, 15, 16, 11, 15, 17, 13, 16, 18, 12, 11, 15, 18, 19, 20, 17, 17, 12, 11 and 15. For the second group of patients, the scores are as follows: 17, 18, 16, 19, 21, 14, 15, 16, 17, 21, while for the third group of patients: 23, 24, 19, 21, 20, 15, 15, 25, 26, 21, 17, 19, 22, 17, 18, 19, 23, 26, 21, 22, 19, 19, 20, 16 and 25 scores. Could you check if there are statistically significant differences between the groups I am comparing?

Sure, to check if there are statistically significant differences between the groups, you can perform an analysis of variance (ANOVA) test. ANOVA is used when you have more than two groups and want to compare the means of these groups to determine if there are significant differences among them.

First, let's calculate the means and standard deviations for each group:

Group 1: Mean = (13 + 15 + 16 + 11 + 15 + 17 + 13 + 16 + 18 + 12 + 11 + 15 + 18 + 19 + 20 + 17 + 17 + 12 + 11 + 15) / 20 = 15.2, Standard Deviation = 2.747

Group 2: Mean = (17 + 18 + 16 + 19 + 21 + 14 + 15 + 16 + 17 + 21) / 10 = 17.4, Standard Deviation = 2.384

Group 3: Mean = (23 + 24 + 19 + 21 + 20 + 15 + 15 + 25 + 26 + 21 + 17 + 19 + 22 + 17 + 18 + 19 + 23 + 26 + 21 + 22 + 19 + 19 + 20 + 16 + 25) / 25 = 20.32, Standard Deviation = 3.309

Next, you can perform the ANOVA test to see if there are significant differences between the groups. The null hypothesis (H0) is that there are no significant differences, while the alternative hypothesis (Ha) is that there are significant differences.

Since you are conducting this study and have the data, you can use statistical software like R or Python to perform the ANOVA test. In R, you can use the `aov()` function, and in Python, you can use the `scipy.stats.f_oneway()` function.

Here's how you can do it in Python:

python   Copy code

```python
import numpy as np
from scipy.stats import f_oneway

# Define the data for each group
group1_scores = np.array([13, 15, 16, 11, 15, 17, 13, 16, 18, 12, 11, 15, 1
group2_scores = np.array([17, 18, 16, 19, 21, 14, 15, 16, 17, 21])
group3_scores = np.array([23, 24, 19, 21, 20, 15, 15, 25, 26, 21, 17, 19, 2

# Perform the ANOVA test
f_statistic, p_value = f_oneway(group1_scores, group2_scores, group3_scores)

print("F-statistic:", f_statistic)
print("P-value:", p_value)
```

The p-value will indicate whether there are statistically significant differences between the groups. If the p-value is less than your chosen significance level (e.g., 0.05), you can reject the null hypothesis and conclude that there are significant differences in life satisfaction scores among the three groups of asthma patients.

Keep in mind that ANOVA assumes the data meets certain assumptions, such as normality and homogeneity of variances. You may also want to consider performing post-hoc tests (e.g., Tukey's HSD test) if the ANOVA results are significant, to determine which specific group(s) differ significantly from each other.

ChatGPT

chat.openai.com

Default (GPT-3.5)

I investigated Il-21[pg/ml] levels in a group of patients with atopic dermatitis before and after the implementation of drug therapy. Twenty patients took part in the study. The results for the first measurement are as follows: 890, 344, 259, 685, 456, 333, 289, 910, 899, 543, 467, 211, 453, 678, 211, 987, 878, 234, 367 and 459, and for the second measurement: 490, 290, 232, 451, 395, 322, 299, 432, 765, 322, 201, 123, 209, 444, 100, 333, 789, 178, 211 and 400. I used the t-student test for dependent samples, which contributed to the fact that I found statistically significant differences between the two time measurements. The results for the second measurement were found to be statistically significant lower. Did I do the analysis well and could you do it again for me? I want to find out if there was a statistically significant change in the second time period.

Based on your description, it seems like you conducted the analysis correctly using the t-test for dependent samples (also known as a paired t-test) to compare the Il-21 levels before and after drug therapy in patients with atopic dermatitis. This is an appropriate statistical test when you have paired data from the same group of individuals at two different time points.

To verify your results and conduct the analysis again, let's go through the steps:

Step 1: State the hypotheses:

- Null hypothesis (H0): There is no significant difference in Il-21 levels before and after drug therapy in patients with atopic dermatitis.
- Alternative hypothesis (H1): There is a statistically significant difference in Il-21 levels before and after drug therapy in patients with atopic dermatitis.

Step 2: Set the significance level (alpha):
You need to decide on the significance level, which is typically set at 0.05 (5%) for most analyses.

Step 3: Perform the t-test:
Using the given data, let's perform the paired t-test using any statistical software or calculator. I'll assume you are familiar with Excel.

In Excel, you can use the T.TEST function. Here's how to do it:

1. Enter the first measurement data in one column (let's say in cells A2 to A21).
2. Enter the second measurement data in another column (let's say in cells B2 to B21).
3. In a new cell, use the T.TEST function: `=T.TEST(A2:A21, B2:B21, 2, 1)`

The `T.TEST` function takes the following arguments: `T.TEST(array1, array2, tails, type)`.

- `array1` and `array2`: The two data sets you want to compare (before and after measurements).
- `tails`: This should be set to `2` for a two-tailed test since we are testing for any significant difference (not specifically higher or lower).
- `type`: Use `1` since you are performing a paired t-test.

Step 4: Interpret the results:
The output of the T.TEST function will give you the p-value. If the p-value is less than your chosen significance level (alpha), typically 0.05, then you reject the null hypothesis and conclude that there is a statistically significant difference between the two time periods.

If the p-value is less than 0.05 (or whatever alpha you chose), then you can conclude that there is a statistically significant change in Il-21 levels during the second time period after drug therapy.

Keep in mind that the smaller the p-value, the stronger the evidence against the null hypothesis, and the more confident you can be in your conclusion.

So, perform the test and check the p-value to determine whether the second time period's results are statistically significant or not.

ANOVA for FeNO Subgroups

chat.openai.com

Default (GPT-3.5)

OR

In an article published in July 2022 in Allergy, the authors compared 3 subgroups of patients divided by fractional exhaled nitric oxide (FeNO). Fifty-one people were included in one group, 46 in the second and 18 in the third. Among other things, the authors made comparisons in terms of eosinophilia counts. What statistical test do you think they could have used to check whether the three groups differed in a statistically significant way?

To check whether the three groups (divided by fractional exhaled nitric oxide - FeNO) differ in a statistically significant way with respect to eosinophilia counts, the authors could have used an analysis of variance (ANOVA) test.

ANOVA is an appropriate statistical test when there are multiple groups being compared and a continuous outcome variable, such as eosinophilia counts in this case. It allows researchers to determine if there are statistically significant differences between the means of the groups. In this study, the three groups represent different levels of FeNO, and the authors want to investigate whether these different FeNO levels are associated with different eosinophilia counts.

If the ANOVA test indicates that there are significant differences among the three groups, the authors can proceed with post-hoc tests (e.g., Tukey's test, Bonferroni correction) to determine which specific groups differ from each other. These post-hoc tests help to identify pairwise comparisons that are statistically different and provide more detailed information on the differences between the groups.

Keep in mind that to use ANOVA, certain assumptions need to be met, such as normality of the eosinophilia count data and homogeneity of variances between the groups. If these assumptions are not met, alternative non-parametric tests like the Kruskal-Wallis test may be considered. However, given that ANOVA is commonly used in medical research and has good statistical power when its assumptions are met, it's a reasonable choice for this type of analysis.

ChatGPT

chat.openai.com

Default (GPT-3.5)

OR

In an article published January 2022 in Allergy, the authors investigated the effect of dupilumab on improving health-related quality of life. This was a phase 3 study of a certain project. The group of patients taking dupilumab included 438 patients, while placebo included 286. The authors observed the presence of statistically significant differences between the groups. Quality of life was assessed using a 22-item sinus-nasal test score. The maximum possible score was 110 points. What measure of effect size do you think they could have used to show the practical significance of the results obtained?

To show the practical significance of the results obtained in the study, the authors could have used a measure of effect size. One commonly used measure of effect size in this type of situation is Cohen's d.

Cohen's d is a standardized measure that quantifies the difference between two means in terms of standard deviation units. It allows researchers to interpret the magnitude of the difference between the treatment group (taking dupilumab) and the control group (placebo) in a standardized way, making it easier to understand the practical significance of the findings.

To calculate Cohen's d, the formula is:

$$d = \frac{\text{Mean}_1 - \text{Mean}_2}{\text{Pooled Standard Deviation}}$$

Where:

- Mean_1 is the mean score of the group taking dupilumab.
- Mean_2 is the mean score of the placebo group.
- The pooled standard deviation is calculated as the square root of the average of the variances of both groups.

By comparing Cohen's d to certain benchmarks, researchers can assess the practical significance of the effect size. As a general rule of thumb, a Cohen's d around 0.2 is considered a small effect, around 0.5 a medium effect, and around 0.8 or higher a large effect.

If the authors used Cohen's d or a similar effect size measure in their study, it would provide a clearer understanding of the magnitude of the differences in health-related quality of life between the dupilumab group and the placebo group, beyond just stating whether the differences were statistically significant.

Small Sample Non-Parametric

chat.openai.com

Default (GPT-3.5)

OR

In a paper published in January 2023 in Allergy, the authors wanted to assess the relationship between IgE levels for peach components and IgE levels for food, inhalant allergens and latex if there were at least 10 patients. What analysis for this type of sample size should they use?

For a study with at least 10 patients, the appropriate statistical analysis would depend on the specific research question, the distribution of data, and the type of variables being measured. Generally, with a small sample size, non-parametric statistical tests are often preferred, as they are less sensitive to the assumptions of normality and can be more reliable with limited data.

In the case of assessing the relationship between IgE levels for peach components and IgE levels for food, inhalant allergens, and latex, where the sample size is relatively small (at least 10 patients), non-parametric tests would be a suitable choice. Here are a couple of options:

1. Spearman's Rank Correlation:
   Spearman's rank correlation is a non-parametric measure of the strength and direction of association between two variables. It is used when both variables are measured on an ordinal or continuous scale. This test will help assess whether there is a monotonic relationship between IgE levels for peach components and other allergens.
2. Mann-Whitney U test (Wilcoxon rank-sum test):
   If the IgE levels for peach components and other allergens are measured for two independent groups (e.g., allergic and non-allergic individuals), the Mann-Whitney U test can be used. It is a non-parametric test that assesses whether there are significant differences in the distribution of IgE levels between the two groups.
3. Kruskal-Wallis test:
   If the study includes more than two groups (e.g., comparing IgE levels for different allergens in multiple patient groups), the Kruskal-Wallis test can be used. It is a non-parametric alternative to one-way ANOVA and can determine whether there are significant differences in the distributions of IgE levels among the different groups.

Remember, the choice of statistical test should be based on the data and the specific research question. If you have access to the actual data and more details about the study design, you can consult a statistician for guidance on the most appropriate analysis.

ChatGPT

chat.openai.com

Default (GPT-3.5)

OR

Published in Allergy in December 2019, the results of the study looked at dual blockade of IL-4 and IL-13 with dupilumab. The authors used a one-way analysis of variance. They mentioned a comparison of standard deviations in independent groups. What F statistics correction should they apply when it would appear that the variances are not homogeneous?

When the variances of groups are not homogeneous (i.e., they are significantly different), the authors should apply a correction to the F statistic. This correction is called the Welch's ANOVA (Analysis of Variance). The traditional one-way ANOVA assumes equal variances across all groups, which is not appropriate when the assumption of homogeneity of variances is violated.

Welch's ANOVA adjusts the F statistic to account for unequal variances and is more robust in such situations. It is generally preferred when the assumption of equal variances is not met. This correction provides a more reliable and accurate test for comparing means of independent groups when the standard deviations differ.

In summary, when the variances are not homogeneous, the authors should use Welch's ANOVA instead of the traditional one-way ANOVA to compare the means of different groups in their study involving dual blockade of IL-4 and IL-13 with dupilumab.

ChatGPT

chat.openai.com

Default (GPT-3.5)

OR

In a letter to the editor published in February 2023 in Allergy, the authors investigated the early increase in serum specific IgG2 after allergen immunotherapy. Measurements were taken before the start of therapy, after 4 months and after 1 year. The group of patients treated with the 300-IR HDM tablet was divided into two subgroups of 25 patients in each group. What test should the authors apply to check for statistically significant differences in each of these subgroups between the three time periods being compared?

To check for statistically significant differences in each of the two subgroups between the three time periods (before the start of therapy, after 4 months, and after 1 year), the authors should apply a repeated measures analysis of variance (ANOVA) test.

Repeated measures ANOVA is used when the same group of participants is measured at multiple time points or conditions. In this case, the same patients are measured at three different time periods. It is an appropriate test for comparing means across multiple related samples, such as pre-treatment, 4 months into treatment, and 1 year into treatment.

The test will help the authors determine if there are significant changes in serum specific IgG2 levels within each subgroup over time. Additionally, it allows for the investigation of any interactions between the treatment (300-IR HDM tablet) and time, providing insights into whether the treatment had a significant impact on the observed changes over time.

By using repeated measures ANOVA, the authors can assess whether the differences in serum specific IgG2 levels between the three time periods are statistically significant for each of the two subgroups separately, providing valuable insights into the effectiveness of the allergen immunotherapy.

ChatGPT

chat.openai.com

OR

I would like to test whether there are statistically significant differences in cough severity in the group of 50 asthmatic patients I studied. Measurements were taken five times during treatment. Each patient could tick a response of strong, moderate, or weak cough severity. Which statistical test should I use?

To test for statistically significant differences in cough severity among the group of 50 asthmatic patients over multiple measurements, you should use a statistical test suitable for analyzing repeated measures or dependent samples. Since you have multiple measurements for each patient, a suitable test would be the "Repeated Measures Analysis of Variance" (RM-ANOVA) or the "Repeated Measures Analysis" (also known as the "within-subjects ANOVA").

The RM-ANOVA is appropriate when you have a single dependent variable (cough severity) measured at multiple time points (five measurements) within the same group of participants (asthmatic patients). It assesses whether there are significant differences in the mean cough severity across the five time points.

Before conducting the RM-ANOVA, ensure that your data meets the assumptions of normality, sphericity, and homogeneity of variance. If your data violates these assumptions, you may need to consider alternative statistical tests or transformation techniques.

If the assumption of sphericity is not met, you could use the Greenhouse-Geisser correction or Huynh-Feldt correction to adjust the degrees of freedom.

Additionally, if you are interested in comparing specific time points against each other, you could perform post hoc tests (e.g., Bonferroni, Tukey, or Sidak) to identify which time points have significant differences.

Remember to set the significance level (alpha) for your test, typically 0.05, to determine the statistical significance.

Overall, by using RM-ANOVA, you can determine if there are statistically significant differences in cough severity among the 50 asthmatic patients across the five measurements during treatment.

McNemar's Test for Cough ×

chat.openai.com

Default (GPT-3.5)

OR

I would like to test whether there are statistically significant differences in cough severity in the group of 50 asthmatic patients I studied. Measurements were taken five times during treatment. Each patient could tick a response of strong, moderate, or weak cough severity. Which statistical test should I use?

Since you have a categorical variable (cough severity) measured at different time points for the same group of individuals, you should use a statistical test that is suitable for analyzing repeated measures or within-subjects designs. One commonly used test for this type of data is the **Chi-squared test for independence**.

However, the Chi-squared test assumes that the data follows a multinomial distribution and that the observations are independent. In your case, since the same group of asthmatic patients is being measured multiple times, the assumption of independence might not hold true. Patients' responses are likely to be correlated across time points within the same individual.

A more appropriate test for this situation would be the **McNemar's test**. McNemar's test is used when you have paired nominal data and want to assess whether there are changes over time. In your study, you have repeated measurements of cough severity within the same patients over time, which fits the criteria for McNemar's test.

McNemar's test is usually applied when you have a 2×2 contingency table of paired nominal data. In your case, you could construct such a table like this:

sql Copy code

```sql
            Before Treatment   Time 1   Time 2   Time 3   Time 4   After Tre
--------------------------------------------------------------------------------
No Change   n1                 n2       n3       n4       n5       n6
Change      m1                 m2       m3       m4       m5       m6
```

Here, "No Change" represents patients whose cough severity didn't change from the previous measurement, and "Change" represents patients whose cough severity changed. You will calculate the McNemar's test statistic from this table.

Make sure you appropriately handle any missing data or incomplete responses in your analysis.

If you have specific statistical software (like R or Python with relevant libraries), you can use functions like `mcnemar.test` in R or `statsmodels.stats.contingency_tables.mcnemar` in Python to perform the test and obtain the p-value for assessing the statistical significance of the differences in cough severity over time.

ChatGPT
chat.openai.com
Default (GPT-3.5)

OR

I would like to test whether there are statistically significant differences in cough severity in the group of 50 asthmatic patients I studied. Measurements were taken five times during treatment. Each patient could tick a response of strong, moderate, or weak cough severity. Which statistical test should I use?

In your study, you are dealing with a categorical variable (cough severity) that has three levels (strong, moderate, weak), and you want to determine if there are statistically significant differences in cough severity over time among a group of 50 asthmatic patients. Since you have multiple measurements for each patient taken at different times, a suitable statistical test to use in this scenario is the Chi-squared test for independence.

The Chi-squared test for independence is commonly used to analyze the association between two categorical variables, in this case, cough severity and time points. It will help you determine if there's a significant relationship between the two categorical variables (cough severity and time) across your sample.

Here's how you can set up your analysis:

1. **Null Hypothesis (H0):** There is no association between cough severity and time points. In other words, the distribution of cough severity is the same across all time points.
2. **Alternative Hypothesis (H1):** There is an association between cough severity and time points. In other words, the distribution of cough severity is different across at least one pair of time points.
3. **Test Statistic:** You will calculate the Chi-squared statistic based on the observed and expected frequencies of cough severity at each time point.
4. **Degrees of Freedom:** The degrees of freedom for the Chi-squared test in this case would be (Number of Rows - 1) * (Number of Columns - 1). Since you have 3 cough severity categories and 5 time points, the degrees of freedom would be (3 - 1) * (5 - 1) = 8.
5. **Significance Level (Alpha):** Choose a significance level (such as 0.05) to determine if the p-value is less than this threshold.
6. **Critical Value or p-value:** Calculate the p-value associated with the Chi-squared statistic. If the p-value is less than your chosen significance level, you can reject the null hypothesis.
7. **Conclusion:** If the p-value is less than your chosen significance level, you can conclude that there is a statistically significant association between cough severity and time points, indicating that the cough severity distribution is not the same across all time points.

Keep in mind that the Chi-squared test assumes certain conditions, such as the independence of observations and expected frequencies being reasonably high. Additionally, if you have specific hypotheses about the trend of cough severity over time, you might also consider using more advanced methods like ordinal logistic regression.

Before performing any statistical analysis, it's a good practice to consult with a statistician or someone experienced in data analysis to ensure that the chosen method is appropriate for your data and research questions.
